# Explaining Variations in Mindfulness Levels in Daily Life

**DOI:** 10.1007/s12671-018-0932-1

**Published:** 2018-03-22

**Authors:** Han Suelmann, André Brouwers, Evelien Snippe

**Affiliations:** 1 Faculteit Psychologie en Onderwijswetenschappen, Open Universiteit NL, Postbus 2960, 6401DL Heerlen, The Netherlands; 2 Interdisciplinary Center Psychopathology and Emotion regulation, Unversity of Groningen, University Medical Center Groningen, Groningen, The Netherlands

**Keywords:** Mindfulness, Attention, Intention, Experiential avoidance, Experience sampling

## Abstract

Despite the apparent benefits of being mindful, people are often not very mindful. There seem to be forces that drive people toward as well as away from mindfulness. These forces are conceptualised in terms of competition for scarce attentional resources. To explore these forces and to test this framework, an experience sampling study was performed among people with an explicit intention to be mindful and an ongoing practice to examine concurrent associations between state mindfulness and daily life experiences that may affect it. Participants (*N* = 29, 1012 observations) filled out questions on momentary experiences at semi-random intervals, five times a day, over a period of 7 to 10 days. Predictors of within-person variations in awareness of Present Moment Experience (PME) and non-reactivity to PME were examined using multilevel analyses. Participants were more aware of PME when they had an activated intention to be mindful and when they felt good, and not very busy or hurried, and were not involved in social interaction. They were more reactive to PME when they experienced unpleasant affect, and when they were hurried or tired. An activated intention to be mindful was also associated with an increased tendency to analyse PME. Experiencing threat was associated with increased reactivity, but not with decreased awareness. Our study generally supports the idea that competition for attention can be a fruitful framework to describe mechanisms behind being or not being mindful.

Why are people, even people having an explicit intention to be mindful, not always mindful? The benefits of mindfulness and mindfulness training have received ample attention in present-day psychological research (e.g. Brown et al. 2007; Keng et al. 2011), yet little is known regarding the mechanisms that enhance or diminish one’s mindfulness in daily life. Insight into these mechanisms would not only be theoretically interesting. It would potentially have applications in further development of mindfulness-based interventions (MBIs). Such knowledge could, for example, be used to educate MBI participants, supporting their intention to be mindful during the day. The learning processes involved in developing mindfulness could be explored, facilitating MBI optimisation to maximise learning and transfer. Besides that, explaining fluctuations in mindfulness might enhance our general understanding of mechanisms underlying attention and awareness.

In their operational definition of mindfulness, Bishop et al. (2004) define mindfulness in terms of not just non-elaborative awareness of current experience but also “an orientation of curiosity, experiential openness and acceptance” (p. 234). Being mindful therefore implies attending to present-moment experience (PME) *itself* rather than shifting attention to *reactions to* PME, such as analysis of its content or attempts to change PME. Reactions to PME can, of course, themselves be experienced, and attending to that *experience* would constitute mindfulness. Building on the definition of Bishop et al., two aspects of mindfulness can be distinguished: *awareness* (of PME) and *non-reactivity*, the latter representing the non-elaborative nature of awareness and an open and accepting orientation. Thus conceptualised, these two aspects of mindfulness are states, not traits.

State non-reactivity should then not be confused with trait acceptance. The latter, as conceptualised by Cardaciotto et al. (2008), has been framed, along with similar measures, as “willingness and readiness to expose oneself to experiences, non-avoidance” (Bergomi et al. 2013, p. 192). (Trait) acceptance may be thought of as the ability and willingness to generally stay out of (state) reactivity in the face of difficult PME.

As the concept of attention is crucial to mindfulness, theoretical models of attention may help understand why and when people are mindful or not. The theoretical framework presented below was inspired by Desimone and Duncan’s (1995) biased competition model of visual attention, as elaborated by Knudsen (2007). This model describes how information competes for access to working memory (i.e. for attention). The most salient information “wins”, which means that it is attended to. Working memory has some control over the competition by modulating the sensitivity to certain kinds of information. Thus, working memory “biases” the competition, hence the name of the model. The framework presented below is not limited to visual attention, and rests upon four principles: (1) competition for attention, (2) partial control over attention, (3) a preference for pleasant experiences and (4) a preference for what is perceived as important.

Principle 1 is the existence of a competition for attention. As it is impossible to attend to everything simultaneously (e.g. Knudsen 2007; Schneider and Shiffrin 1977), possible objects of attention compete for allocation of attentional resources. Such possible objects of attention include various kinds of PME (such as sensory impressions), as well as, for example, goal pursuit and thought content. (Distinguishing between attending to the *content* of thought (i.e. thinking) and attending to the *experience* of thought, the former does not qualify as mindfulness, the latter does.) The strongest competitor “wins” the competition. It follows that one’s degree of mindfulness critically depends on the strength of PME in that competition. This does not imply that mindfulness is incompatible with attending to things like work or socialising. Yet, it does seem to imply that, for example, attending to a deadline can attract attention so completely that the connection with PME is lost.

The existence of a competition for attentional resources implies that generic “busyness” (such as doing one’s work or chores) is associated with decreased awareness. After all, resources needed to attend to PME are not available when one is very busy with other tasks that require attention. Furthermore, the intention to be mindful may be rapidly forgotten in demanding situations, just like the intention to perform some action may be rapidly forgotten in such circumstances (Einstein et al. 2003).

Principle 2 is that attention is partially under (conscious) control (Knudsen 2007). An activated intention to attend to some object will increase the likelihood of attending to that object. One can consciously bring attention to an object, but objects—especially salient objects, such as the pain when one’s finger is hit with a hammer—can also attract attention apart from (or even against) such regulation.

One may thus consciously aim to be mindful. A general intention to be mindful will not always be on the top of one’s mind. The level of its activation varies between situations and will affect one’s degree of mindfulness. The importance of intention for being mindful has been stressed before by Shapiro et al. (2006). (They, however, include intention in their *conceptualization* of mindfulness, rather than identify intention as a variable *affecting* mindfulness.)

Principle 3 is that a pleasant experience is more motivating to attend to than an unpleasant one. The tendency to avoid “problematic” experiences has been called *experiential avoidance* in the context of psychopathology (Hayes et al. 1996). However, it seems to be relevant outside that context, and to be “a basic component of the human condition” (Hayes et al. 1996, p. 1155). Pleasant PME is, therefore, more likely to be attended to than unpleasant PME. Unpleasant experiences may also trigger mental processes searching for ways to change that experience for the better, such as rumination and distraction seeking. (In terms of competition for attention, reactiveness to PME competes with PME itself for attention.) Consistent with this, unpleasant affect was found, in an experimental setting, to increase the tendency toward mind wandering (Smallwood et al. 2009).

On the level of trait—rather than state—variables, Moore et al. (2009) found a negative correlation between experiential avoidance and mindfulness. Several MBIs aim (among other things) to decrease experiential avoidance, and were indeed found to do so, with the expected associations between changes in experiential avoidance and changes in mindfulness (Kearney et al. 2012). Fatigue and loss potential should also decrease awareness of PME and increase reactivity, because of their unpleasantness. Apart from being unpleasant, fatigue may also be associated with a general reduction of attentional resources, leading to less awareness of PME.

Principle 4 is that attention tends be allocated to things considered important, in the sense that they have the potential to bring rewards or threaten to bring about some kind of loss. A lot of attention research was done in the context of visual attention, and the effects of rewards have been researched extensively (Chelazzi et al. 2013). Not only do rewards directly motivate attention, signals that have been rewarded in the past can also involuntarily attract attention, even if these signals are no longer associated with rewards (Chelazzi et al. 2013). Loss potential presumably has at least equally strong—and probably even stronger—effects, as there is a large body of evidence showing that humans are “programmed” to attend to threats, even more than to rewards (Baumeister et al. 2001).

This suggests that issues involving loss potential decrease awareness of PME. This applies, among other things, to things threatening to go wrong, or being in a hurry to the point of perceiving a threat of being late. It also suggests that their inherent unpleasantness will increase reactivity. Social interaction may also compete with PME for attention, because of the strong reward and loss potential associated with it.

The goal of the current study was to test our framework by examining whether the mechanisms described above are supported by real-life within-person correlation patterns. Since competition for attention occurs between possible objects of attention that appear at the same moment in time, all hypothesised effects are (near-) instantaneous. Therefore, the focus was on examining concurrent associations among state mindfulness and daily life experiences that may affect it. The hypotheses to be tested were that (1) individuals are less aware of PME when they are busy; (2) there is (a) more awareness and (b) less reactivity when there is an activated intention to be mindful; (3) there is (a) more awareness and (b) less reactivity when one’s affect is perceived pleasant; (4) more fatigue is associated with (a) less awareness and (b) more reactivity; (5) in situations involving loss potential, there is (a) less awareness and (b) more reactivity; and (6) during social interaction, there is less awareness.

## Method

### Participants

Participants of the study were 30 individuals (29 Dutch, 1 Belgian) with meditation experience in traditions related to and valuing mindfulness, such as vipassana and Mindfulness-Based Stress Reduction (MBSR; Blacker et al. 2009). The inclusion criteria were having an explicit intention to be mindful, and currently having a meditation practice in a tradition valuing mindfulness, not necessarily MBSR or Mindfulness-Based Cognitive Therapy (MBCT; see Segal et al. 2002). We considered an intention to be mindful important, because people without a *general* intention to be mindful are unlikely to have the *activated* intention to be mindful that figures in hypothesis 2. Participants were recruited using sign-up lists (at vipassana retreats and a mindfulness teachers meeting, *N* = 11), direct participation requests (aimed at specific people presumed to satisfy the entry criteria, mainly because of participation in retreats, MBSR, or mindfulness teacher training programme, *N* = 11) and snowballing (*N* = 8). Two additional individuals who did not meet the inclusion criteria were excluded before data were gathered. One other participant was dropped from the data analyses after data gathering (see the “Results” section). Participant demographics are presented in Table 1, omitting the one dropped participant.

**Table 1:** Demographic characteristics of participants

	Mean	SD
N	29	–
Age	47.7	9.3
Males	21%	–
Meditation experience (year)	6.8	4.9
Practice (h/week)	4.0	2.4
Reports/participant	34.9	9.1
Response rate	79%	13%
Current practice^a^		
MBSR	15	
Vipassana	13	
Yoga	8	
Ridhwan	2	
Zen	1	
Other	2	
Total Vipassana and/or MBSR	83%	

^a^Because some participants reported more than one practice, the numbers do not add up to the total number of participants

### Procedure

All items, as well as procedures and tools, were tested in a small pilot study in a convenience sample of five participants; none of them participated in the main study. Participants were interviewed to determine how they had interpreted the items. The items used to assess mindfulness were found to be unsuitable to measure state mindfulness. They were therefore replaced by a new scale, subjected to further development and validation steps, as described in more detail in the next subsection. No problems surfaced regarding the other items.

Participants were invited to participate in an experience sampling study. Experience sampling has the distinct advantage of enabling the examination of within-person, across-occasion variation in real life (e.g. Palmier-Claus et al. 2011).

Participants received written instructions and an explanation of the questionnaire items. They were then interviewed to ascertain that they met the entry criteria and that they understood the instructions and the questionnaire items. They were explicitly instructed to ask, rather than guess, if there was any doubt regarding items or procedures during the research period. The interview was also used to “snowball” for more participants, and to gather demographic data, such as age, sex, meditation experience and current meditation practice.

The experience sampling schedule involved a period of 7 to 10 days, depending on participant preference and willingness. Five times a day, at semi-random intervals, between 8.45 AM and 9.45 PM, participants received a signal prompting them to answer the questions. Participants could either fill out the questions online on their smartphone or by paper and pencil, depending on their own preferences and (phone) hardware possession. Participants in the smartphone group (*N* = 26) received messages from the SurveySignal software (Hofmann and Patel 2014) on their smartphones, prompting them to fill out the questionnaire. By clicking on a link in the message, participants accessed the online questionnaire to answer the questions. Participants in the paper-and-pencil group (*N* = 3) received SMSs (following semi-random schedules similar to those of the smartphone group), prompting the answering of the questions in a paper-and-pencil format.

Participants were not to enter data after more than 15 min unless they could recall the moment of the signal accurately, for example when they had noticed the signal but were unable to answer immediately. Following McCabe et al. (2012), responses not entered within an hour after the signal were excluded. However, differing from their approach, such responses were blocked at the data gathering stage—rather than sifted out at the time of analysis—for the smartphone group.

Two days after the start of the data collection, participants were approached to verify that data collection was on the right track, and to check whether there were issues or questions (following the recommendations of Palmier-Claus et al. 2011). After the data collection period, participants had a debriefing interview to identify any issues and to record any other relevant remarks. Participants were rewarded by providing them the results and conclusions when the study was finished. They were also given the opportunity to opt-in for feedback regarding their own correlation patterns, though with caution, and without making any between-participant comparisons.

#### Instrument Development and Validation of the Mindfulness Scales

Existing state mindfulness scales were not suitable for the present study for several reasons. The State MAAS (Brown and Ryan 2003) is based on a very different conceptualisation of mindfulness; it measures general awareness, rather than awareness of PME, and does not include items assessing non-reactivity. The Toronto Mindfulness Scale (TMS) was developed “in reference to an immediately preceding mindfulness meditation session” (p. 1462), and therefore, its validity may not be generalizable to mindfulness in daily life (Lau et al. 2006; see also Thompson and Waltz 2007). The State Mindfulness Scale (SMS; Tanay and Bernstein 2013) comprises 21 items, and is therefore too long to be used in experience sampling research.

An attempt to derive a shorter scale from the SMS was unsuccessful. In the first pilot study with five participants (none of them participating in the main study), some items proved very sensitive to circumstances not directly related to mindfulness. For example, an item intended to measure noticing of bodily sensations proved very sensitive to the *presence* of bodily sensations. Furthermore, non-reactivity is not represented in the SMS, limiting content validity for the purposes of the present study. Because of these issues with existing mindfulness scales, we decided to construct a new measure capturing state awareness of PME and non-reactivity to PME, suitable for use in experience sampling.

To construct a new questionnaire, a preliminary set of eight items was created. Because of our conceptualisation of mindfulness, the questionnaire covered questions measuring both awareness and non-reactivity. Two items assessing awareness were formulated, reflecting awareness of PME and attending to PME. Six preliminary items were formulated to assess non-reactivity, reflecting the non-elaborative, open and accepting orientation to PME. They included various ways of being reactive (e.g. seeking distraction). Because the items effectively assessed ways of not being mindful, they were all reverse-coded. The way in which participants comprehend and respond to these items was examined using a method inspired by Belzer et al. (2013). A convenience sample of 14 participants (including all five participants of the first pilot) responded to the new mindfulness items while thinking aloud, answering the questions as pertaining to the last thing they did before the interview (which was conducted by phone). They were instructed to say what they were thinking, describing all considerations, questions, memories and reasoning that occurred in their minds in response to the items.

Some modifications were made at this stage. The noun “experience” (Dutch: “ervaring”) was sometimes interpreted as “past experience”, and was therefore eliminated from the wordings of some items. The item “I am interested in what I experience” was dropped because it tended to be interpreted in terms of active analysis of PME. The item “I am attending more to my experience itself than to my reactions to that experience” was dropped because it led to confusion, especially among experienced meditators, who felt that this distinction could not be made. One item was rephrased: “I know what’s going on inside me” was replaced by “I am aware of what’s going on inside me”. The resulting six items were found to be comprehensible and unambiguous, and were interpreted as intended.

To investigate the items in an experience sampling setting, a subsequent second small pilot study (four participants, a subset of the participants of the first pilot) was performed, which focused on the mindfulness items. The items were found to have good interpretability and applicability to real-life situations, and no further changes were made.

In the final analysis, the item assessing absorption (“I'm so consumed by one thing that everything else passes me by”) was dropped because it did not fit well into the factor structure of the mindfulness scales. The final set of five items (see the “Appendix” section) had a factor structure of two factors (judged by the scree plot), clearly identifiable as awareness and non-reactivity, with all items loading on the intended scales. The *analysis* item (“I am analysing what I experience”) cross-loaded somewhat on the awareness scale, which makes sense, as will be discussed below. The final set of items is listed in the “Appendix” section.

### Measures

Because the participants had to fill out the same questionnaire many times, the questionnaire had to be short in order to promote compliance (Palmier-Claus et al. 2011). Therefore, the constructs were examined with only a few questions. Moreover, Schimmack and Grob (2000) found that items measuring directly constructs such as affect pleasantness and fatigue have very high factor loadings (0.83–0.90), which indicates that even a single item can measure such a construct in a fairly accurate way.

#### Mindfulness: Awareness and Non-Reactivity

We aimed to measure two aspects of mindfulness: *awareness* and *non-reactivity*. Awareness was measured with two items (example: “I am aware of what goes on inside me”), α = 0.87. Non-reactivity was measured with three items (example: “I resist what I am experiencing”, reverse coded), α = 0.75 (see the “Appendix” section).

#### Activated Intention to Be Mindful

Activated intention to be mindful was measured with three items, pertaining to situational cues, momentary awareness of intention and trying to be mindful, respectively (α = 0.80). Example: “I am trying to be mindful right now”.

#### Loss Potential

Three items were constructed to measure loss potential. As discussed below, analyses showed that the three items had quite different correlation patterns. Consequently, the scale was split into two scales, named “hurry” (single item: “I’m at risk of being late.” and “threat” (two items, example: “Something is threatening to go wrong.”), α = 0.76.

#### Social Interaction

Social interaction was measured with two items, assessing being with others and communicating with others, respectively, α = 0.80. (Example: “I am alone”, reverse scored.)

#### Fatigue

Fatigue was measured with a single item: “I am tired”.

#### Affect Valence

Affect valence was measured with a single item: “I feel good”.

#### Busyness

Busyness was measured with a single item: “I am very busy”.

Except for the items assessing social interaction, 5-point Likert scales were used, ranging from 1 (not at all) to 5 (fully). The items assessing social interaction were rated on a three-point scale, anchored “No”, “More or less” and “Yes”, scored 1, 3 and 5, respectively, to have consistent ranges for all items in the questionnaire. Scale scores were computed by averaging item scores. The items that were used in the questionnaire were in Dutch and are listed in the “Appendix” section. The English translations of the items have not been validated for use in questionnaires.

### Data Analyses

The data have a hierarchical structure, with reports (level 1) clustered within participants (level 2). Multilevel regression analysis takes this structure into account (e.g. De Leeuw and Meijer 2008). Multilevel restricted maximum likelihood regression analyses were performed using the lmer function of the R package lme4 (version 1.1-7; Bates et al. 2015).

Several multilevel regression analyses were performed. First, to examine each variable’s relationship with mindfulness, a series of univariable multilevel analyses were performed with each individual variable separately as a predictor, and awareness or non-reactivity as the outcome. The independent variables were the ones specified in the hypotheses. Second, we performed a series of multivariable multilevel analyses in which predictors of mindfulness were entered simultaneously. To account for a possible effect of awareness on non-reactivity, awareness was included as a predictor in the multivariable multilevel analysis of non-reactivity. Additionally, multilevel analyses were performed to verify the effects of predictors on each other that were mentioned in the introduction (e.g. threat, being unpleasant, leading to decreased affect valence).

Standard deviations, correlation coefficients and Cronbach alphas were computed after centring around participant means. This ensures that between-participant variance is removed.

Because we expected that all hypothesised effects are (near-) instantaneous, only concurrent and no time-lagged associations were examined. We expected mindfulness levels to vary rather quickly, and computed lagged correlations to verify this.

Random effects were initially included for all independent variables, thus allowing the relationships between variables to vary between participants. The purpose of this was to ensure accurate prediction of average effects. The random effect of hurry on non-reactivity was omitted because it caused convergence problems, because the effect was small and because adding the random effect did not improve model fit according to Akaike’s (1973) information criterion.

All planned hypothesis tests were one-tailed, *α* = 0*.*05. All reported *p* values are one-tailed, unless stated otherwise. If, in a report, a single item was missing from a scale consisting of more than two items, the scale score was still computed as the mean of the available items. All analyses were then performed on an available-case basis and missing data were not imputed.

The multilevel analyses are based on maximum likelihood methods that rely on normality and homoscedasticity assumptions (Van der Leeden et al. 2008). To check for problems caused by possible violations of these assumptions and prevent inflated type-1 error rates, we performed case resampling bootstrap analyses of the multilevel multiple regressions with awareness and non-reactivity as dependent variables. Bias-corrected confidence intervals and significance levels for correlations were estimated by a case resampling bootstrap at participant level, creating 10,000 resampled datasets for each analysis. Bootstrap methods may yield unbiased estimates of standard errors when maximum likelihood methods do not (Van der Leeden et al. 2008). Similar to the main analyses, random effects were originally included for all independent variables. To remedy problems regarding convergence and performance, all random effects that did not improve model fit according to Akaike’s information criterion were removed from the bootstrap computations.

As there were no trends apparent in the data, we did not remove linear trends from the data.

## Results

### Data Cleaning, Preparation and Characteristics

One participant from the paper-and-pencil group produced answers that generally seemed erratic. The numbers of signals per day were inconsistent with the signals that were actually sent. The participant cancelled the debriefing and did not respond to subsequent attempts to contact her. Her data were therefore excluded from the analysis.

During the debriefing, it became clear that one participant misinterpreted two items of the awareness scale and one participant misinterpreted one item of the activated intention scale; these were treated as missing data for these participants. Overall, 0.5% of the data was missing at item level and 0.4% at variable level in the available reports. In total, 1012 reports were available for analysis (response rate 79%).

Median response time was 3 min; 69% of the answered (smartphone) signals were answered within 15 min. A comparison of quick answers (within 15 min) with slow answers (not within 15 min) yielded no statistically significant differences in the correlation coefficients of both mindfulness dimensions with their predictors, *χ*^2^(14) = 12.1, *p* = 0.60.

The means and standard deviations of the variables under study are presented in Table 2. As is evident from Table 2, some of the variables have skewed distributions. In particular, participants reported on average low levels of hurry and threat, close to the minimum values.

**Table 2:** Descriptive statistics

Variable	Mean	Within-person SD	Total SD
Awareness	3.11	0.87	0.93
Non-reactivity	4.37	0.62	0.79
Activated intention	2.70	0.91	1.15
Hurry	1.33	0.72	0.81
Threat	1.23	0.59	0.66
Social interaction	3.30	1.59	1.68
Affect valence	3.27	0.83	1.06
Fatigue	2.74	0.94	1.23
Busyness	2.85	1.10	1.27

### Predictors of Awareness and Reactivity

**Fig. 1:**
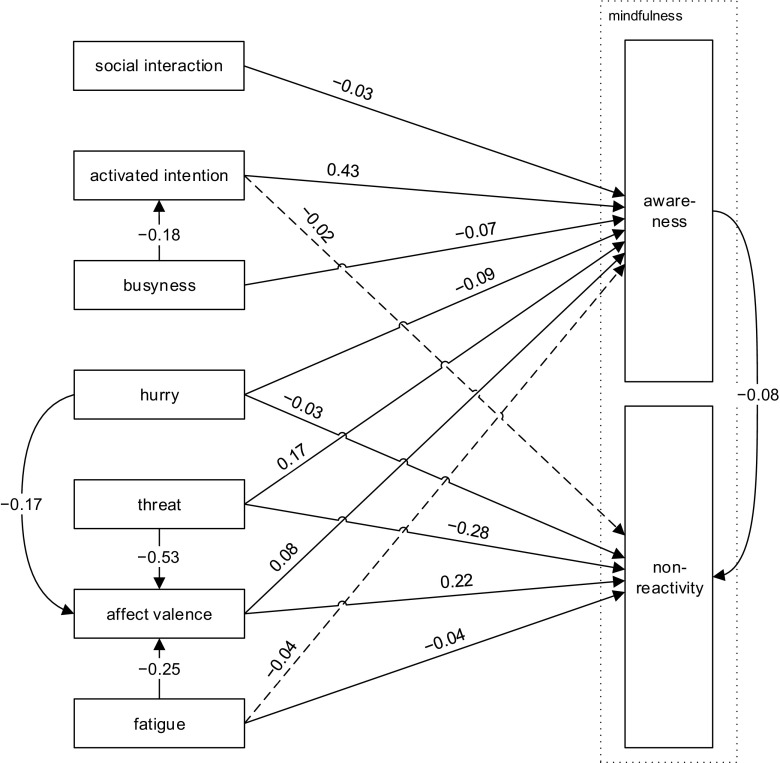
Results of multilevel (multivariable) regression analyses. Unstandardized regression coefficients having one-tailed *p* < 0.05 represented by solid lines, those having *p* > 0.05 by dashed lines

The results of the univariable multilevel analyses predicting either awareness or non-reactivity are presented in Table 3. The results of the multivariable multilevel analyses are depicted in Fig. 1.

**Table 3:** Results of the univariable multilevel analyses: individual predictors of awareness (*N* = 28) and non-reactivity (*N* = 29)

	Awareness			Non-reactivity		
	*B*	SE	*p*	*B*	SE	*p*
Activated intention	0.472	0.055	0.000	− 0.062	0.044	0.079
Hurry	− 0.208	0.059	0.000	− 0.085	0.036	0.008
Threat	0.037	0.085	0.332	− 0.449	0.072	0.000
Social interaction	− 0.031	0.024	0.095			
Affect valence	0.177	0.066	0.004	0.282	0.048	0.000
Fatigue	− 0.062	0.041	0.067	− 0.135	0.030	0.000
Busy	− 0.179	0.039	0.000			
Awareness				− 0.082	0.024	0.000

All significance levels are one-tailed

*B *= unstandardized regression coefficient, *SE* = standard error

As hypothesised (Hypothesis 1), people were less aware when they were very busy. This was shown both in the univariable analysis and in the multivariable analysis.

As predicted (Hypothesis 2a), people were more aware of PME when they had an activated intention to be mindful. In terms of regression coefficients, this is the strongest relationship that was found.

In contrast with our expectations (Hypothesis 2b), having an activated intention was not associated with non-reactivity. To examine this further, separate regression analyses of the individual items of non-reactivity were performed. Activated intention to be mindful was associated with increased analysis of PME, *B* = 0.189, SE = 0.059, *p* < 0.01 in a univariable multilevel regression; for the other two non-reactivity items, no significant relationship was found. To explore the possibility that the increased analysis of PME was associated with increased awareness of PME, the analysis was repeated, including awareness as an additional predictor. This indeed reduced the association between activated intention to be mindful and increased analysis of PME to being statistically non-significant, *B* = 0.089, SE = 0.056, *p* (two-tailed) = 0.12.

As predicted (Hypothesis 3), people tended to be more aware and less reactive when feeling good. Both effects are evident in the univariable analysis and the multivariable analysis.

In the univariable as well as the multivariable analyses, the association between fatigue and awareness was negative, as expected (Hypothesis 4a), but did not reach statistical significance. As predicted, fatigue was associated with increased reactivity to PME (Hypothesis 4b).

The associations of hurry and threat were not as simple as suggested by Hypothesis 5. Hurry and threat were differently associated with awareness and non-reactivity. Being in a hurry was associated with lower levels of awareness of PME, but the association with non-reactivity to PME was rather weak (see Table 3 and Fig. 1). In contrast to this, threat was not associated with awareness in the univariable analysis (Table 3) and was positively associated with awareness in the multivariable analysis (Fig. 1). Also, threat had a strong negative association with non-reactivity in both the univariable and multivariable analyses.

When people were in social interaction, they were less aware of their present moment experience, as predicted (Hypothesis 6), controlling for other predictors (Fig. 1). The univariable regression yielded a relationship of essentially the same strength, though in that analysis, the association was not statistically significant.

### Bootstrap Analyses

The bootstrap analyses, each keeping 10,000 resampled datasets with good convergence, yielded results similar to the original multilevel analyses. The same regression coefficients were significant as in the multilevel regression analyses. This was true for the multiple regression analyses of awareness as well as non-reactivity.

### Relationships Among Predictors

Additional multilevel analyses were performed to examine relationships between predictors. The results are included in Fig. 1. To verify that hurry, fatigue and threat tend to be experienced as unpleasant, a multilevel regression analysis was performed with these three variables as predictors, and affect valence as the outcome. All three variables were indeed found to be negatively associated with affect valence. The relationship of threat with affect valence was much stronger than that of hurry.

Furthermore, a regression analysis with busyness as predictor and activated intention to be mindful as outcome was performed. The expected negative association was indeed found.

### Affect Valence

The predicted relationship between affect valence and awareness, though in the expected direction and statistically significant, was somewhat smaller than expected. This raised the suspicion that it may be smaller because participants who are avoiding an unpleasant feeling are unlikely to report (or even notice) that feeling. To explore this possibility, a subset of the data was created, containing the data of only the experienced meditators. “Experienced” was operationalised as having at least 3 years of meditation experience, having a current practice of at least 3 h a week and having silent retreat experience. The reasoning behind this was that these indicators signal intensive practice, associated with a more developed ability to detect “hidden” feelings. Eighteen participants fulfilled these criteria. The hypothesis was that experienced meditators would be more able to detect repressed unpleasant affect while filling out the questionnaire, thus avoiding the attenuation of the relationship between affect valence and awareness. Indeed, for experienced meditators, there was a considerably stronger association between affect valence and awareness (*B* = 0.162, *SE* 0.045, *p* < 0.01) compared with less experienced meditators (*B* = − 0.040, *SE* 0.059, *p* = 0.25). The difference is statistically significant (Δ*B* = 0*.*202, *SE* 0.074, *p* < 0.01).

### Mindfulness Levels in Consecutive Reports

Changes in mindfulness levels were quick; mindfulness levels in one report were hardly correlated with mindfulness levels in the next report on the same day (for the same participant), *r*^2^ = 0.015 for awareness and *r*^2^ = 0.030 for non-reactivity, indicating that no more than 3% of the variance is explained by the previous report.

## Discussion

This study focused on explaining within-person variations in awareness of Present Moment Experiences (PME) and non-reactivity to PME. Associations between variations in momentary mindfulness and other variables throughout the day were examined. Generally speaking, real-life momentary mindfulness levels indeed varied in a way that is consistent with the proposed principles.

Our results support the prediction that people would be less aware of PME when they are very busy (Hypothesis 1). This was the most direct prediction following from competition for attention (Principle 1); it therefore provides a direct corroboration of our theoretical framework.

Having an activated intention to be mindful was associated with increased awareness of PME (Hypothesis 2a), corresponding with the principle that attention is partially under conscious control. In contrast with our expectations, an activated intention to be mindful is associated with increased analysis of PME, which is a form of reactivity to PME. A plausible explanation for this finding is that awareness of PME is necessary in order to analyse it. If the intention to be mindful increases awareness, it thereby facilitates analysis of PME, a possibility also suggested by the cross-loading of the analysis item (of the non-reactivity scale) on the awareness factor. This explanation is supported by the finding that the association between having an activated intention to be mindful and non-reactivity is non-significant in the multivariable analysis, where awareness is also included as a predictor.

Third, pleasant feelings were hypothesised to increase awareness and decrease reactivity. The results showed that participants were indeed more aware and less reactive when they felt good (Hypotheses 3a and 3b). This is consistent with the results of Brown and Ryan (2003), who found that affect was positively associated with mindfulness both between and within subjects.

Participants were less aware when they were tired (Hypothesis 4a). However, the relationship between fatigue and awareness was only marginally statistically significant. Participants also reported higher levels of reactivity to PME when they were more tired, as expected (Hypothesis 4b). Considering the strong relationship between fatigue and affect valence, it seems plausible that individuals react to fatigue because it they experience it as being unpleasant.

Regarding Hypothesis 5 (the effect of loss potential), the results suggest that hurry and threat have markedly different effects. Being in a hurry was associated with reduced present moment awareness. This association may arise because hurry, which is often associated with a need for immediate action, reduces mindfulness by competing for attention. Threat, on the other hand, was mainly associated with increased reactivity. This may be caused by threat being an unpleasant experience. The difference in the effects of hurry and threat is consistent with our finding that affect valence has a stronger relationship with threat than with hurry. The difference is also consistent with the examples participants gave of typical situations in which they experienced hurry or threat. These examples mostly implied that hurry required immediate action, such as needing to arrive in time or getting a job done quickly. For threat, participants mostly described unpleasant situations beyond their immediate control, such as interpersonal issues or impending lack of income.

Threat, by itself, was not associated with awareness. However, when analysed alongside our other predictors, it was associated with increased awareness, instead of decreased awareness. The difference between the univariable and multivariable analyses may arise because when threat and affect valence are entered into the same regression analysis, any effect of threat mediated by affect valence is absorbed into the regression coefficient of affect valence, and only a direct effect remains. It is possible that our participants, intending to be mindful and practicing to that end, may have developed a “reflex” to be aware of PME when difficulties arise. Unfortunately, this speculation could not be tested with our dataset. Our findings do not imply that threat increases mindfulness; on the contrary, threat is strongly associated with increased reactivity to PME.

Participants were less aware when they were involved in social interaction (Hypothesis 6). This corroborates the prediction that, because social interaction is demanding and associated with considerable reward/loss potential, it claims scarce attentional resources, diminishing awareness of PME. The effect was, however, rather weak. It is not clear to what extent our participants’ social interactions differ from those of the general population or how this effect size generalises to the general population. Plausibly, our selected group may have more “mindful” contacts, reducing the tendency of their social interactions to decrease mindfulness.

An interesting question is whether the statistical relationships reflect causal effects on awareness and reactivity. Our findings corroborate the hypothesised causal effects in a limited sense, as they generally support the testable hypotheses derived from them, but the possibility that mindfulness affected the predictors under study, rather than the other way around, cannot be excluded. The associations under study were concurrent associations, and therefore do not contain information on the direction of the effects.

The question of causality is especially pertinent with respect to the effect of affect valence on mindfulness. Previous findings suggest that mindfulness and affect valence influence one another mutually (Gotink et al. 2016; Snippe et al. 2015). An experience sampling study by Gotink et al. (2016) supports our hypothesis that affect valence influences state mindfulness as they found that momentary affect predicted mindfulness at a later moment. (Their mean time between measurements is smaller in their study than ours, enabling them to find such relationships.) Consistent with our findings, Gotink et al. also found that mindfulness was correlated with concurrent affect, showing a positive correlation with positive affect and a negative correlation with negative affect.

In contrast, Snippe et al. (2015) did not find evidence for a lagged effect of negative and positive affect on mindfulness. However, due to their long time lag (1 day), they may have missed within-day lagged effects of affect on mindfulness. Furthermore, they did not measure non-reactivity, and the current study showed especially strong associations between affect valence and non-reactivity. The results of Snippe et al. (2015) supported an effect in the opposite direction by showing that increases in daily mindfulness were followed by lower levels of negative affect and higher levels of positive affect the next day.

### Limitations

The sample in this study was a selected group, consisting of individuals with an explicit intention to be mindful and with meditation experience. Though not all participants were currently practicing MBSR/MBCT and/or vipassana, the large majority did. Because of this selection, the results may not generalise to the general population. Future studies may address the applicability of the framework of competition for attention in individuals without intention to be mindful or meditation experience. It may well apply to individuals intending to be mindful, but with no—or less—meditation experience. It seems unlikely, however, that the presence of an activated intention to be mindful is a major factor in individuals not intending to be mindful. It seems likely that, without the driving force of intention, there will not be much mindfulness at all, and even if there is, it will not be sustained for long. It is in this sense that we agree with Shapiro et al. (2006) that intention to be mindful can be regarded as a crucial component of mindfulness.

Other limitations apply to the measurement of variables. The research is based on self-report, and experience sampling severely restricts the number of items in the questionnaire. The mindfulness items that were used in the questionnaire do not capture subtle nuances such as mindful observation of reactivity, reducing content validity somewhat. On the other hand, existing (trait) mindfulness scales have been criticised because of issues regarding differential understanding of items (e.g. Grossman 2011). The use of experience sampling reduces such issues, because our analyses are based on within-participant, rather than between-participant variance. Furthermore, the way in which the items were understood was validated before the main study, and participants were given explanations and ample opportunity to ask questions.

A limitation inextricably connected with experience sampling is that what remains unconscious tends to go unnoticed (and unreported). The decision to specifically study participants practicing to be mindful, supposedly being relatively aware, was intended to counter that limitation.

Another limitation of the study is, as touched upon earlier, that inferences about the direction of the associations cannot be made, as concurrent associations were examined. However, as the hypotheses suggest that the experiences would have an almost instant effect on mindfulness, examining time-lagged associations would not solve this issue.

Furthermore, our data on threat may have mainly reflected situations in which the threat was relatively obvious, hence the low average of loss potential, though the latter could also be explained by threat (and hurry) being relatively rare). However, the items had sufficient within-person variance to be able to find relationships.

### Future Research

Questions regarding mechanisms affecting state mindfulness have not been explored much; thus, numerous issues still need to be investigated. First, there is no reason to presume that the proposed principles provide a complete description of the relevant mechanisms. Future research may identify other mechanisms affecting mindfulness. For example, the present study is limited to variables exhibiting considerable variance over a period of (at most) 10 days. Thus, factors such as long-term learning and habit formation could not be detected, nor have they been elaborated on in the proposed theoretical framework. Put differently, changes in *trait* mindfulness (viewed as time-averaged state mindfulness) could not be detected. Given the propensity of the human brain to learn by forming habits (e.g. Wood and Neal 2007), it is plausible that competition for attention and being mindful will also be heavily influenced by habit formation. Furthermore, the present study addressed the effects of loss potential. However, our framework also predicts that *reward* potential affects mindfulness. Future studies may address this.

Second, the importance of an *activated* intention to be mindful demonstrates the importance of intention to be mindful per se. Therefore, in-depth research on the determinants of intention to be mindful is warranted. The Theory of Planned Behavior (Ajzen 1991; Ajzen and Fishbein 1980) could be a fruitful starting point for this type of research. Furthermore, the importance of having an intention to be mindful suggests that MBIs might benefit from aiming at developing and sustaining that intention. The development of such an intention is, of course, not foreign to MBIs in their current form, but putting more effort into supporting participants in their intention to be mindful, may enhance this crucial ingredient of the development of mindfulness. Future research may aim at finding effective ways to do this.

Third, affect was simplified to a single-item, one-dimensional construct (i.e. its valence). Yet, affect has been found to have two (e.g. Watson and Tellegen 1985) or even more (Gilbert et al. 2008; Schimmack and Grob 2000) dimensions. It is not a priori clear that all these dimensions should have identical relationships with mindfulness. As an example, the factor labelled “activated positive affect” by Gilbert et al. may be associated with reward pursuit. Signalling this, two of the items that make up this factor are “excited” and “eager”. Theoretically, reward pursuit is expected to *decrease* mindfulness. Such nuances were not measured in the present study, but could be examined in future studies measuring affect in a more detailed way.

Fourth, future research may explore the relationship with Self-Determination Theory (e.g. Ryan and Deci 2002). That theory assumes that there are basic psychological needs, and that their fulfilment is important. Olafsen (2017) hypothesised that insufficient need satisfaction may therefore cause the mind to wander to causes and/or solutions for the dissatisfaction, thereby reducing mindfulness. Her reasoning seems to fit well within a framework regarding competition for attention. She found that need support at work and need satisfaction are indeed associated with increased mindfulness. In a similar vein, a lack of need fulfilment in general might be associated with being less mindful.

Fifth, the results suggest that specific factors (busyness, social interaction, threat, hurry, unpleasant affect) are especially challenging for someone trying to be mindful. This suggests that specifically practising under these difficult circumstances could optimise transfer of what has been learned to daily life.

To remind participants of their intention to be mindful and to practice at moments that being mindful is especially challenging, the “Stop, Breathe and Be” exercise of the .b (“dot-be”) MBI for adolescents (Burnett et al. 2011) might be helpful. It involves participants sending each other short text messages at random moments, which then trigger the recipient to do a short meditative exercise (S. Hennelly, personal communication, Oct. 25, 2016). This reminds the recipient of the intention to be mindful, and promotes practicing at random moments, including difficult circumstances as well. Otherwise, attempts to be mindful may well be more or less limited to convenient, relatively easy, circumstances. This is borne out both by the (negative) association between busyness and activated intention to be mindful, as found in the present study, and Einstein et al.’s (2003) finding that “forgetting intentions in demanding situations is rapid”. The effects of this exercise—or similar ones—may be verified in future research.

## Appendix

### Experience Sampling Questionnaire Items

- A1 Ik ben me bewust van wat er in me omgaat. (I am aware of what goes on inside me.)
- A2 Ik heb aandacht voor wat ik ervaar. (I pay attention to what I am experiencing.)
- N1 Ik verzet me tegen wat ik ervaar. (I resist to what I am experiencing.)
- N2 Ik zoek afleiding. (I look for distractions.)
- N3 Ik ben mijn ervaring aan het analyseren. (I’m analysing my experiences.)
- I1 Ik ben me bewust van mijn intentie mindful te zijn. (I am aware of my intention to be mindful.)
- I2 Ik probeer nu mindful te zijn. (I am trying to be mindful right now.)
- I3 De situatie herinnert me aan mijn intentie mindful te zijn. (The situation reminds me of my intention to be mindful.)
- L1 Ik dreig te laat te zijn. (I am at risk of being late.)
- L2 Er dreigt iets mis te gaan. (Something is threatening to go wrong.)
- L3 Er dreigt iets akeligs te gebeuren. (Something nasty is threatening to happen.)
- P1 Ik voel me prettig. (I feel good.)
- F1 Ik ben moe. (I am tired.)
- B1 Ik ben druk bezig. (I am very busy.)
- S1 Ik communiceer met anderen. (I am communicating with others.)
- S2 Ik ben alleen. (I am alone.)

Items A* make up the awareness scale. Item N* make up the non-reactivity scale. Items I* make up the *activated intention* scale. Items L* make up the *Loss Potential* scales (L1 = hurry; L2 and L3 = threat). Items S* make up the *Social interaction* scale. P1, F1 and B1 assess affect valence, fatigue and busyness, respectively.
